# The Effect of Modification on the Properties of Polyetherimide and Its Carbon-Filled Composite

**DOI:** 10.3390/polym12051056

**Published:** 2020-05-04

**Authors:** Azamat Slonov, Ismel Musov, Azamat Zhansitov, Elena Rzhevskaya, Diana Khakulova, Svetlana Khashirova

**Affiliations:** Kabardino-Balkarian State University Named after H.M. Berbekov, st. Chernyshevsky, 173, 360004 Nalchik, Russia; azamatslonov@yandex.ru (A.S.); ismel@mail.ru (I.M.); elena.r-1382@mail.ru (E.R.); dianakhakulova@mail.ru (D.K.); new_kompozit@mail.ru (S.K.)

**Keywords:** polyetherimide, polycarbonate, polyphenylene sulfone, carbon fibers, modification, mechanical properties, 3D printing

## Abstract

The effect of oligophenylene sulfone (OPSU) and polycarbonate (PC) on the rheological, mechanical and thermal properties of polyetherimide (PEI) and a carbon-filled composite based on it was studied. It is shown that the introduction of OPSU and PC leads to a decrease in the melt viscosity of PEI and a carbon-filled composite based on it with the preservation of their mechanical properties and heat resistance at a sufficiently high level. It was found that composites with OPSU have higher mechanical and thermal properties compared with composites with PC. Samples from modified carbon-filled PEI were printed by the fused deposit modeling (FDM) method. Three-dimensionally printed samples from carbon-filled PEI modified by OPSU showed significantly higher mechanical properties than composites with PC.

## 1. Introduction

One of the classes of polymers used in various branches of modern technology with increased requirements for mechanical properties and thermal stability are high performance polymers. In recent years, these polymers have come to the fore as promising matrices for composite materials. High-performance polymers and composites based on them, in addition to increased strength and heat resistance, also have resistance to shock cyclic loads and cracking, high atmospheric and chemical resistance [1], which, in some cases, allows them to be used instead of metals [2].

Moreover, these materials are of significant interest to one of the fastest growing industries as additive manufacturing or 3D printing. Fused deposit modeling (FDM) is the most widely used 3D technology for polymer materials. Most of the work in the field of 3D printing by FDM is devoted to the study of standard and engineering plastics [3], such as acrylonitrile butadiene styrene (ABS) [4,5], poly-lactic acid (PLA) [6,7], nylon [8], polycarbonate (PC) [9,10], as well as their combinations [11,12,13], but high performance plastics are also actively explored in 3D printing [14,15,16]. 

The enhanced properties of high-performance polymers are ensured by their rigid chain structure and strong intermolecular interactions. On the other hand, these features greatly complicate their processing and obtaining composite materials based on them. A particularly difficult task increasing their manufacturability, which provides processing by high-speed injection molding and 3D printing methods for the manufacture of large-sized products of complex design with reduced wall thickness.

At present, the most promising amorphous high-performance thermoplastic used in 3D printing is polyetherimide (PEI). It is amorphous thermoplastic with high thermal stability and remarkable mechanical properties. It is used, among other applications, for advanced parts in electrical and electronics industry, in aircraft applications and in the automotive industry [17]. 

Basically, works on 3D printing of PEI are related to the study of the influence of printing parameters, such as a temperature, an angle of raster laying, an air gap between rasters, a width of the rasters, etc., on mechanical properties of samples from this material. For example, the effect of printing parameters on the mechanical properties of PEI was studied in [18]. It was shown that the raster orientation and nozzle temperature were closely related to the mechanical properties of printed samples. At the nozzle, the temperature is 370 °C, and 0° raster orientation reached mechanical properties close to the injection-molded parts. In [19], the influence of printing parameters on the mechanical properties of samples was also studied, where Stratasys PEI ULTEM 9085 was used. This material is a blend of PEI with PC. It is shown that the highest properties are achieved when using a negative gap between rasters. Moreover, in [20], the same material was used for study and it was found that the most optimal combination of properties is achieved with an air gap of 0 mm and a raster angle of 0°. In [21], the authors carried out the modification of PEI by PC and compared with the properties of the industrial brand for printing ULTEM 9085. It was revealed that the introduction of PC leads to an increase in the rheological properties of PEI. The viscosity of the obtained composite with a content of 10% PC is close to the viscosity of the industrial brand, while exceeding it in strength and elasticity modulus.

However, of particular interest for 3D printing are composites based on PEI with carbon fibers (CF), since they are characterized by high mechanical properties [22,23,24] and can be used for aircraft detail printing. In particular, [25] shows the results of printing PEI with a content of 10 % CF, compared to the ULTEM 9085. Both materials have been used to print aircraft engine components. It is shown that the printed samples of the carbon-filled composite are inferior in properties to the samples obtained on the basis of industrial material. The authors consider the reason for this to be the porosity and increased viscosity of the carbon-filled material.

It is known that the introduction of fibrous fillers significantly increases the polymer melt viscosity [26,27]. When processing by injection molding, this leads to underfilling and defective products, and, when processed by 3D printing (FDM), to poor adhesion of the filaments in the sample and, as a result, to low mechanical properties [28]. Moreover, 3D printing demands that the materials have higher rheological properties than injection molding does. The fact is that the melts of polymer materials, being non-Newtonian fluids, change viscosity depending on shear rates—as a rule, the higher the shear rate, the lower the melt viscosity. It is known that 3D printing takes place at very low shear rates [29,30], when the melt has the highest viscosity; therefore, materials for 3D printing should have very high rheological properties.

It is possible to achieve high melt flow in two ways—by increasing the processing temperature or by introducing modification additives into the composition of the material. Printing of polymeric materials by the FDM method takes place at temperatures significantly higher than injection molding temperatures; therefore, a decrease in the melt viscosity of fiber-filled composites by increasing the already high printing temperatures will lead to the destruction of the material. The most optimal way to achieve the necessary rheological properties is the introduction of modification additives into the polymer material.

The existing industrial plasticizers and lubricants on the market have low thermal properties and are mainly used for standard plastics with low processing temperatures [31,32,33] and are therefore unsuitable for plasticizing high-performance polymers. This served as the basis for the study of the effectiveness of modifiers for fiber-filled composite materials based on PEI intended for 3D printing.

## 2. Materials and Methods 

The object of the study was PEI Ultem 1010 manufactured company Sabic (Saudi Arabia) as a matrix polymer. An oligomer of polyphenylene sulphone (OPSU) with the following characteristics was used as a modifier: a molecular weight (MW) of about 19,000, an onset of destruction temperature of 470 °C and a glass transition temperature of 190 °C. It was synthesized according to the method described in [34] using 4,4′-dihydroxy diphenyl and 4,4′-dichloro diphenyl sulfone. The polycarbonate (PC) of the K-20 MRA brand of the Carbotex company (Japan) was used as modifier either. It has a temperature of the onset of destruction at about 430 °C and a glass transition temperature of 145 °C. As a filler, milled carbon fibers with a length of 0.2 mm and diameter of 7 micrometers from R&G (Germany) were used.

**Figure 1:**
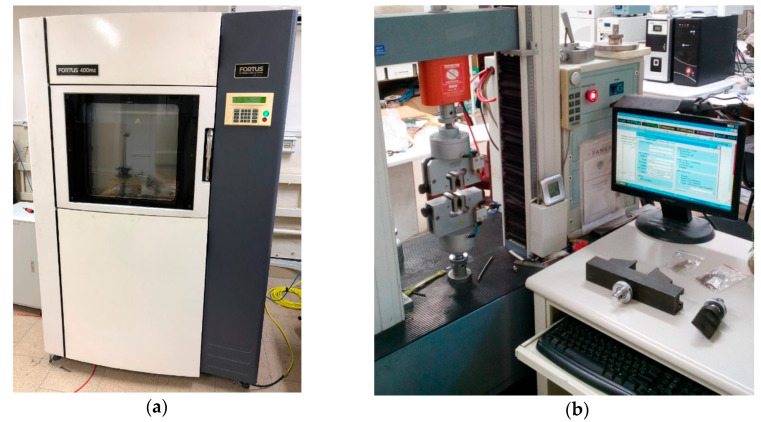
3D printer Fortus 400mc (**a**) and universal testing machine GT-TCS 2000 (**b**).

First, starting components were dried under vacuum at temperatures of 120–170 °C and mixed on a high-speed mixer. The dry mixture was processed at temperatures of 310–360 °C on a TwinTech twin-screw microextruder (UK) with L/D = 30. Test samples were obtained by injection molding on an SZS-20 machine from Haitai Machinery (China) at a material cylinder temperature of 340–380 °C and a mold temperature of 180 °C. Standard test samples were printed on a Stratasys Fortus 400mc 3D printer (Figure 1a) at 416 °C.

Rheological properties were determined by melt flow rate (MFR) on an IIRT-5 device (Russia) at a temperature of 350 °C and a load of 5 kg, as well as using a LCR 7001 capillary rheometer from Dynisco (USA) at a temperature of 380 °C. Heat resistance was determined by the method of thermogravimetric analysis (TGA) on a TGA 4000 device from PerkinElmer (USA), at a heating rate of 10 °C/min in air. Glass transition temperature was determined by the method of differential scanning calorimetry (DSC) on a DSC 4000 device from the same company at a heating rate of 10 °C/min in air. Microscopic studies were carried out on a Vega 3 scanning electron microscope (SEM) from Tescan (Czech Republic).

The uniaxial tensile mechanical tests were carried out on dog-bone samples with dimensions according to GOST 112 62-80 type 5. The tests were carried out on a universal testing machine Gotech Testing Machine GT-TCS 2000 (Taiwan) at a temperature of 23 °C (Figure 1b). Impact tests were performed with and without notch, according to the Izod method according to GOST 19109-84 on a Gotech Testing Machine, model GT-7045-MD (Taiwan) with a pendulum energy of 11 J. The average fiber lengths in composite materials were determined according to GOST R 57730-2017.

## 3. Results and Discussion

**Figure 2:**
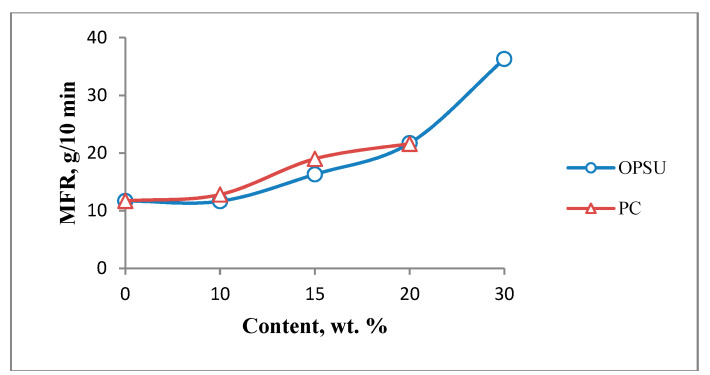
Dependence of melt flow rate polyetherimide (MFR PEI) on the content of oligophenylene sulfone (OPSU) and polycarbonate (PC).

At the first stage, the effect of OPSU and PC on the main properties of unfilled PEI was studied. OPSU was introduced into PEI in the amount of 10, 15, 20, 30 wt.%, and PC 10, 15, 20 wt.%. The study of the rheological properties of PEI after the introduction of modifiers shows (Figure 2) that both OPSU and PC lead to a uniform increase in MFR, with a slight advantage for the values for composites with PC. 

**Figure 3:**
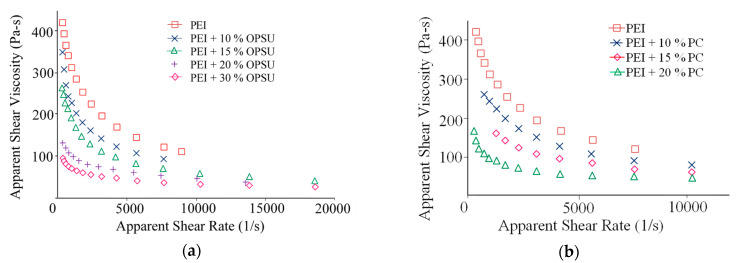
The dependence of the PEI melt viscosity on the content of OPSU (**a**) and PC (**b**).

The plasticization of PEI is also observed on flow curves, where a decrease in the viscosity of the PEI melt with an increase in the content of OPSU and PC is seen (Figure 3a,b). It is noteworthy that the flow curves of composites with an equal content of OPSU and PC are similar, i.e., both materials provide effective plasticization of PEI, and equally.

Table 1 shows that the introduction of OPSU leads to a significant decrease in impact strength, both for notched and unnotched specimens. An increase in the content of OPSU to 30% led to a slight decrease in the strength and elastic modulus of PEI. In this case, the elastic modulus, both in bending and in tension, remains at a sufficiently high level, regardless of the content of OPSU. Upon reaching 20% content, there is a lack of tensile yield strength, i.e., brittle destruction begins.

For composites with PC, a different picture is observed. The introduction of up to 20% PC into PEI leads to a slight increase in toughness and plasticity. The elastic modulus and strength also decrease slightly and remain at a high level.

**Figure 4:**
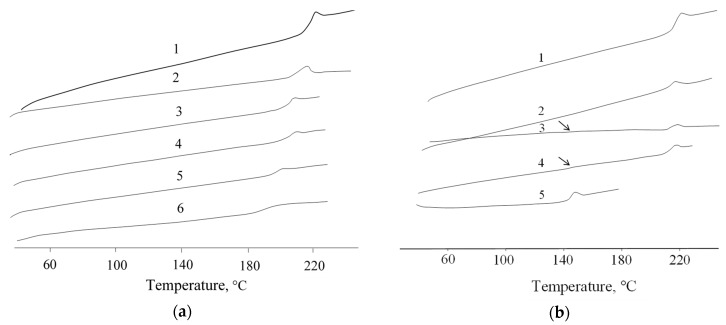
Differential scanning calorimetry (DSC) curves of blends PEI/OPSU (**a**): 1—PEI; 2-5—PEI+10, 15, 20, 30% OPSU, respectively; 6—OPSU and PEI/PC (**b**): 1—PEI; 2-4—PEI+10, 15, 20% PC, respectively; 5—PC.

DSC studies of the obtained blends showed that the introduction of OPSU leads to a uniform decrease in the glass transition temperature (T_g_) of PEI (Figure 4a; Table 2) and only single thermal effects corresponding to the glass transition temperature of PEI are observed. In the case of introducing 10% PC into PEI, a slight decrease in the T_g_ of PEI is also observed; however, a further increase in its content practically does not affect the value of T_g_ (Table 2). On the DSC curve of the composite with 15 % PC content, a second barely noticeable peak is observed in the T_g_ region of the PC (Figure 4b, see arrows), which indicates a forming biphasic structure. Similar patterns were obtained in [21,35]. At 20% content, the peak becomes more pronounced.

**Figure 5:**
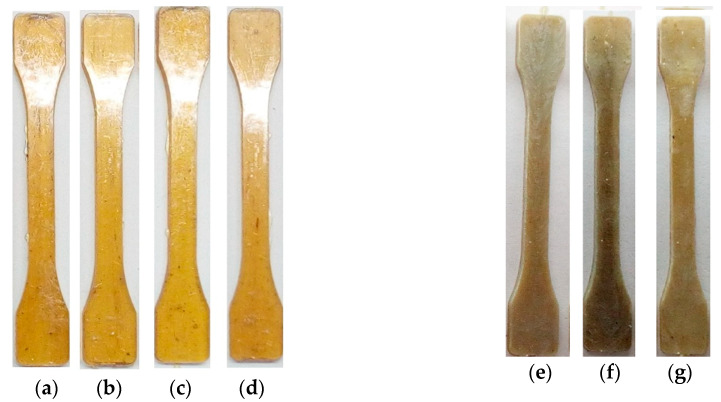
Samples from composites PEI/OPSU (**a**–**d**) and PEI/PC (**e**–**g**).

The immiscibility of PEI and PC is also confirmed by the fact that PEI/PC samples are opaque (Figure 5e–g), while the samples of PEI/OPSU mixtures remain optically transparent over the entire content range (Figure 5a–d), which highlights the good compatibility of these materials. 

(TGA) shows that the introduction of both OPSU and PC into PEI leads to a slight decrease in its heat resistance (Table 3). In the case of using OPSU, a decrease in thermal properties is insignificant since the oligomer itself is characterized by high heat resistance. More noticeably, the thermal stability of PEI decreases with the introduction of PC; however, even in this case, the blends have sufficient resistance to thermal degradation.

Therefore, comparative studies of OPSU and PC as PEI modifiers showed their rather effective and equal plasticizing ability. The high mobility of macromolecules and the fluidity of the OPSU and PC melts at the PEI processing temperature lead to the effect of temporary plasticization, i.e., facilitating the flow of PEI melt. At room temperature, in view of the high glass transition temperatures, both OPSU and PC are in a glassy state, which allows for preserving the elastic modulus and strength properties of PEI. At the same time, PC, due to its high molecular weight and plasticity, promotes the elasticization of PEI. OPSU, on the contrary, having a low MW, leads to embrittlement of the samples.

To study the effect of modifiers on the properties of composites, compositions with a content of 30% carbon fibers (PEI-CF) were used, since, at this content, the MFR significantly decreases, and, at the same time, the composite has rather high mechanical properties [36]. Modifiers were introduced in amounts of 10, 15, 20 wt.% of the polymer content in the composite.

**Figure 6:**
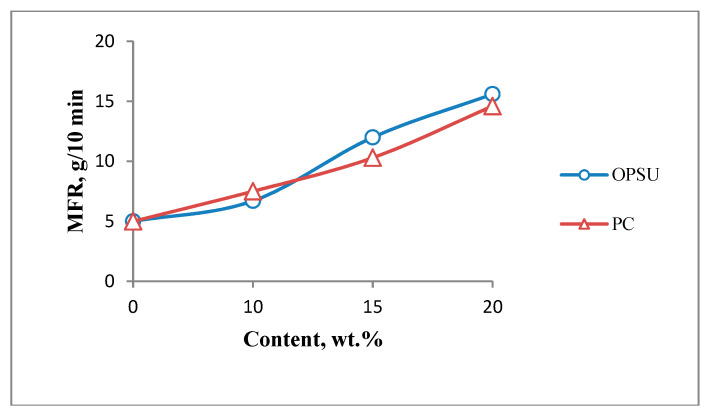
MFR PEI-carbon fibers (CF) with different content of OPSU and PC.

Figure 6 shows the dependences of the MFR of a composite with 30% CF on the content of OPSU and PC. The graph shows that both modifiers equally and quite effectively increase the melt flow of the composite. At a 20% content of OPSU, the MFR increases by 212%, and at a similar content of PC, by 192%.

**Figure 7:**
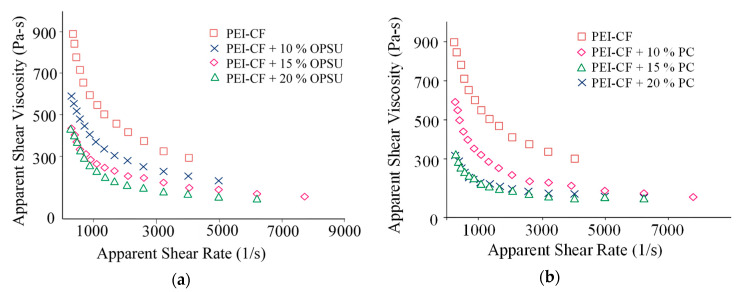
Dependence of the PEI-CF melt viscosity on the content of OPSU (**a**) and PC (**b**).

The study of rheological properties using a capillary rheometer confirmed a decrease in the viscosity of carbon-filled PEI with the introduction of OPSU and PC (Figure 7). The flow curves are similar in nature and viscosity values for equal modifiers content. Apparently, the mechanism of the plasticization of the composite is similar to unfilled PEI.

The mechanical properties of composites containing various quantities of OPSU and PC were also studied to determine their effect on them. Table 4 shows that the introduction of OPSU affects the impact strength of the composite slightly; there is a slight decrease in the impact strength of the composite during tests of unnnotched samples and an increase during the testing of notched samples. In this case, a very pronounced increase in the elastic modulus and strength is observed, both in bending and in tension. The introduction of PC noticeably caused a more significant decrease in toughness and elongation, while there is a significant increase in the elastic modulus. However, unlike composites with OPSU, the bending and tensile strength remains practically unchanged.

The observed changes in the mechanical properties of the carbon-filled composites differ from the effect of these modifiers on unfilled PEI (Table 1), where a greater decrease in toughness and elongation was observed when using OPSU. Apparently, the compatibility of PEI and OPSU in the case of a carbon-filled composite plays a more noticeable positive role, which leads to the production of a composite with higher impact strength and bending and tensile strength, while the heterophasic structure in the case of using PC leads to a lower impact strength.

It is known that, in the process of processing composite materials containing fibrous fillers, there is a significant destruction of the fibers and a reduction in their length, and, consequently, the reinforcing ability [37]. The determination of the residual linear particle sizes of carbon fibers showed that, after extrusion without the addition of a modifier, the particle sizes are reduced by more than 2 times (Table 5). However, it is seen that the introduction of OPSU leads to a greater preservation of fiber lengths; the residual length of CF in composites with the content of OPSU significantly exceed the particle sizes in the composite without modifier. In this case, there is a tendency to produce a slight increase in particle sizes with an increase in the content of OPSU. The introduction of a PC also leads to an increase in size, but to a lesser extent.

Thus, a decrease in the melt viscosity leads to a decrease in shear stresses during processing, which reduces the degree of destruction of the filler particles. Apparently, this leads to a significant increase in mechanical properties with the introduction of OPSU. It is also possible to increase the wettability of the filler particles by a less viscous polymer melt and to improve their adhesive interaction due to the compatibility of OPSU and the polymer matrix, as confirmed by electron microscopy studies.

**Figure 8:**
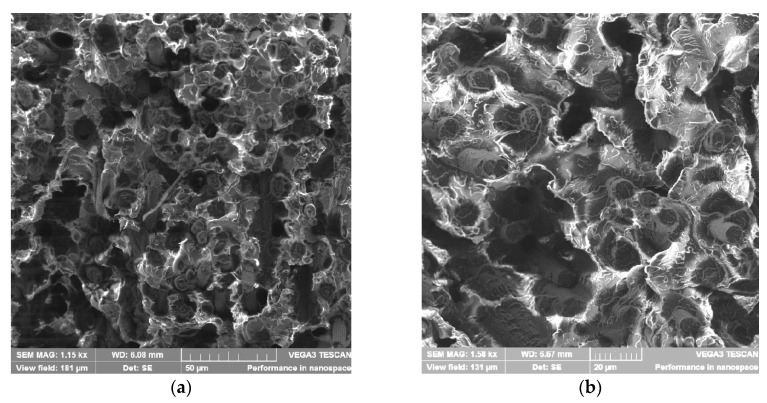

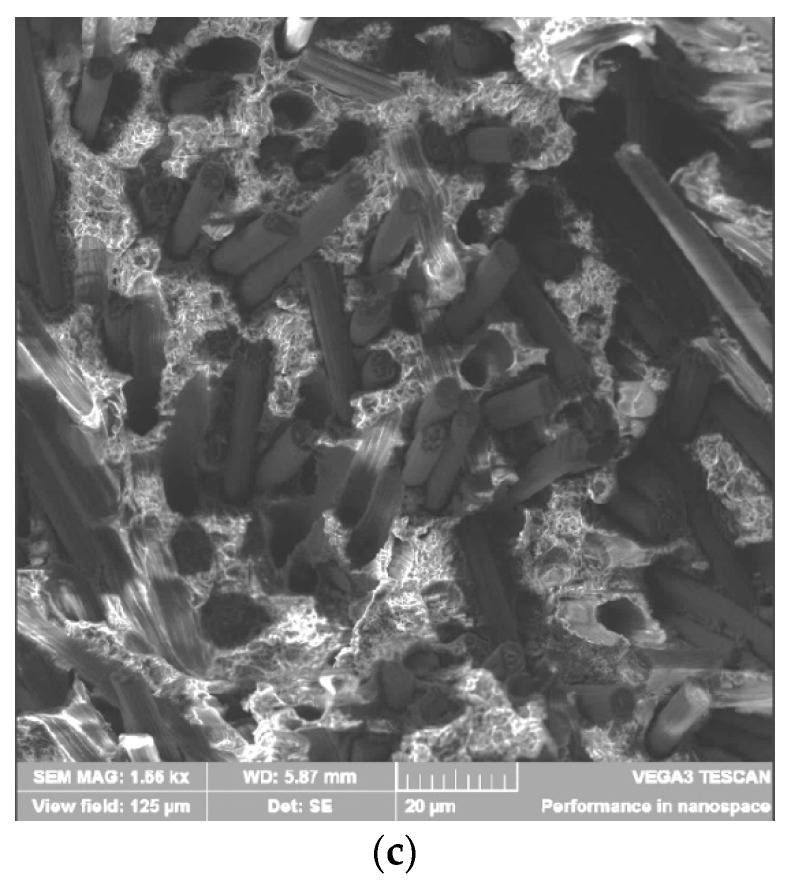
Scanning electron microscope (SEM) images of the broken surface of carbon-filled samples: (**a**)—PEI-CF; (**b**)—PEI-CF + 15% OPSU; (**c**)—PEI-CF + 10% PC.

**Figure 9:**
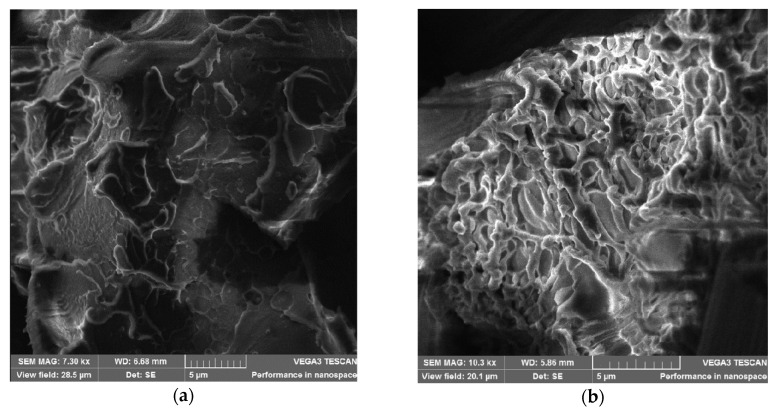
SEM images of the polymer matrix of carbon-filled samples: (**a**)—PEI-CF + 15% OPSU; (**b**)—PEI-CF + 10% PC.

As can be seen in microphotographs, the number of holes from the pulled-out fibers on the surface of the fracture of the specimen with the content of OPSU is noticeably smaller; moreover, a denser fixing of the polymer matrix on the CF surface is observed (Figure 8a,b). In the case of using a PC, on the contrary, there are obvious gaps between the fibers and the polymer matrix (Figure 8c). In this case, one can notice the inhomogeneous structure of the matrix with PC (Figure 9b), in contrast to the mixture with OPSU (Figure 9a), which once again confirms the biphasic structure of PEI and PC mixture and, on the contrary, the miscibility of PEI and OPSU. Apparently, these reasons are associated with a decrease in toughness with the introduction of PC and the absence of an increase in strength properties.

The thermal stability of carbon-filled composites with different contents of modifiers was studied by the TGA method. Heat resistance was evaluated by temperature loss of 2%, 5% and 10% of the mass. Table 6 shows that OPSU practically does not affect the temperature of the destruction of composites, with the exception of the temperature of loss of 2% of the mass at a 10% content of modifier. It should be noted that the temperature of the loss of 2% of the mass can be affected by various random factors, and they are not always regular, in contrast to the temperatures of loss of 5 and 10% of the mass, which are more correct indicators of destruction. In these cases, it is seen that the temperatures are equal to the values of the initial composite—the difference is within the error of the method.

However, it can be noted that PC clearly reduces the temperature of destruction of the material, which is probably due to its lower heat resistance compared to PEI and OPSU. Despite this, the heat resistance of composites with PC content remains quite high.

The thermo-oxidative stability of composites with modifiers was also studied by capillary viscometry. The essence of the study was to measure the melt flow rate of composites at various holding times in the viscometer cylinder at a temperature of 350 °C. As can be seen from Table 7, the starting polymer has a high thermal stability of the melt. The MFR value decreases insignificantly—after holding for 60 min, the MFR decrease is only 10%, which fits into the framework of the method error. The melt stability of the composite with 30% CF also remains quite high—there is no significant change in the MFR.

At 10% OPSU concentration, the MFR practically does not change from the exposure time—the melt is quite stable. However, at higher modifier contents, a noticeable decrease in the MFR with time is observed (by 54% and 63% at 15% and 20% content and exposure time of 60 min). The data obtained indicate a most likely degree of crosslinking with the participation of low molecular weight OPSU, which leads to an increase in melt viscosity.

The introduction of PC leads to a noticeable decrease in the thermal stability of the melt. This temperature is high enough for PC; therefore, as can be seen from Table 7, prolonged aging leads to a significant reduction in the MFR—a reduction that is greater than that of composites with OPSU.

**Figure 10:**
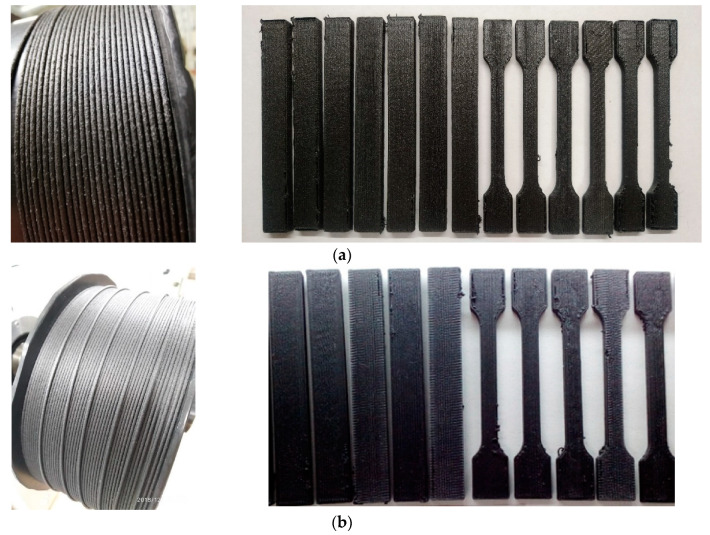
Composite filament for printing and printed test specimens: PEI/CF/OPSU (**a**); PEI/CF/PC (**b**).

Based on these results, a filaments were obtained for 3D printing from composites contenting 25% of CF and 20% OPSU and the same composition but with PC instead OPSU (Figure 10a,b). Standard samples were printed by the FDM method with the longitudinal laying of filaments (filament orientation angle 0 °) and a negative gap between them.

As can be seen from the test results (Table 8), the printed samples have high mechanical properties. However, the composite with OPSU significantly exceeds the similar composite with PC in flexural and tension elastic modulus by 58% and 39%, respectively, and in flexural strength by 117%.

## 4. Conclusions

Thus, it was shown that the introduction of OPSU and PC leads to a decrease in the melt viscosity of PEI. At the same time, the elastic modulus and the strength of PEI are preserved; however, in the case of using OPSU, the toughness is significantly reduced. It was found that PEI and PC form a biphasic structure, while PEI and OPSU are thermodynamically compatible, which is confirmed by DSC, electron microscopy, and the appearance of the samples.

It was revealed that PC and OPSU also significantly reduce the melt viscosity of a carbon-filled composite based on PEI, acting as a temporary plasticizer. In the case of using OPSU, there is a significant increase in the elastic modulus and strength, as well as the conservation of impact strength. The introduction of PC, on the contrary, leads to a decrease in toughness, while the elastic modulus and strength remain at the level of the initial composite.

It is shown that the introduction of PC and OPSU into a carbon-filled composite leads to an increase in carbon fiber length in comparison with a composite without modifiers. Moreover, in the composites with OPSU, a higher adhesive interaction of the polymer matrix with the surface of the carbon fiber is observed. The thermal stability and heat resistance of the melts of composites with OPSU significantly exceed composites with PC.

The printed samples of carbon-filled PEI with OPSU and PC have high mechanical properties; however, the composite with OPSU significantly exceeds the properties of the composite with PC in elastic modulus, both in bending and in tension, as well as in flexural strength.

## Figures and Tables

**Table 1:** Mechanical properties of the blends PEI/OPSU and PEI/PC.

Content	Impact Strength, kJ/m²		E_fl_,	σ_fl_,	E_ten_,	σ_ten_,	σ_yield_,	ε,
	Unnotched	Notched	GPa	MPa	GPa	MPa	MPa	%
PEI	75.5 ± 7.6	6.1 ± 0.6	3.63 ± 0.16	112.4 ± 4.5	2.85 ± 0.08	88.0 ± 5	111.0 ± 5	22.7 ± 11.2
**OPSU**								
10%	39.2 ± 3.9	1.5 ± 0.2	3.29 ± 0,08	104.0 ± 6.7	2.80 ± 0.1	84.0 ± 1.7	106.9 ± 2.8	13.7 ± 2.6
15%	29.7 ± 3.0	1.5 ± 0.2	3.26 ± 0.14	104.1 ± 7.3	2.82 ± 0.08	78.0 ± 0.5	105.2 ± 0.4	12.0 ± 3.7
20%	27.9 ± 2.8	1.2 ± 0.1	3.18 ± 0.1	103.3 ± 9.8	2.81 ± 0.06	103.8 ± 3.2	-	8.5 ± 1.4
30%	7.3 ± 0.7	1.3 ± 0.1	3.27 ± 0.5	65.1 ± 5.3	2.69 ± 0.08	71.4 ± 9.3	-	4.1 ± 2.5
**PC**								
10%	55.7 ± 5.6	4.2 ± 0.4	3.40 ± 0.38	104.2 ± 4.4	2.79 ± 0.09	80.0 ± 0.3	102.5 ± 0.8	15.0 ± 4.6
15%	79.5 ± 8.0	4.6 ± 0.5	3.36 ± 0.13	106.7 ± 8.3	2.77 ± 0.05	81.0 ± 0.5	100.3 ± 0.5	25.0 ± 13.5
20%	84.7 ± 8.5	4.1 ± 0.4	3.28 ± 0.08	100.8 ± 7.7	2.7 ± 0.01	78.0 ± 1.3	96.7 ± 2.9	30.0 ± 5.6

E_fl_: flexural modulus, σ_fl_: flexural strength, E_ten_: tensile modulus, σ_ten_: tensile strength, σ_yield_: yield point, ε: elongation at break.

**Table 2:** Dependence of the glass transition temperature of PEI on the content of OPSU and PC.

Content	T_g_, °C	
	Peak 1	Peak 2
PEI	216.8	-
OPSU	190.0	-
PEI +10% OPSU	211.8	-
PEI +15% OPSU	205.9	-
PEI +20% OPSU	206.4	-
PEI +30% OPSU	198.3	-
PC	145.6	-
PEI +10% PC	214.2	-
PEI +15% PC	214.2	143.0
PEI +20% PC	214.2	142.7

**Table 3:** Thermogravimetric analysis (TGA) data of PEI with the various content of OPSU and PC.

Content	T_2%_	T_5%_	T_10%_
PEI	513.3	530.7	539.3
OPSU	472.0	500.0	523.0
PEI +10% OPSU	507.8	521.8	530.9
PEI +15% OPSU	503.2	521.0	532.0
PEI +20% OPSU	496.1	518.4	528.7
PEI +30% OPSU	498.0	518.6	529.5
PC	432.0	459.0	472.0
PEI +10% PC	492.0	507.0	519.7
PEI +15% PC	483.2	498.0	512.3
PEI +20% PC	480.8	499.5	510.1

**Table 4:** Mechanical properties of carbon-filled PEI with different content of OPSU and PC.

Content	Impact Strength, kJ/m²		E_fl_,	σ_fl_,	E_ten_,	σ_ten_,	ε,
	Unnotched	Notched	GPa	MPa	GPa	MPa	%
PEI-CF (PEI+30% CF)	40.0 ± 4.0	5.6 ± 0.6	12.1 ± 0.4	257.2 ± 5.3	8.2 ± 0.3	154.0 ± 8.2	4.2 ± 0.3
**OPSU**							
10%	38.1 ± 3.8	6.5 ± 0.7	14.4 ± 0.4	256.3 ± 4.6	8.6 ± 0.6	178.5 ± 5.1	4.3 ± 0.5
15%	36.6 ± 3.7	6.3 ± 0.6	16.5 ± 0.4	302.3 ± 6.8	9.7 ± 0.4	191.6 ± 12.1	4.1 ± 0.4
20%	36.1 ± 3.6	6.7 ± 0.7	17.8 ± 0.5	306.3 ± 7.5	8.9 ± 0.3	191.0 ± 7.2	3.8 ± 0.6
**PC**							
10%	20.6 ± 2.1	5.4 ± 0.5	18.6 ± 1.6	264.0 ± 8.1	10.6 ± 0.9	151.0 ± 19.3	2.8 ± 0.5
15%	24.2 ± 2.4	6.0 ± 0.6	18.7 ± 0.3	239.8 ± 4.9	9.7 ± 1.2	157.5 ± 10.3	2.8 ± 0.5
20%	22.7 ± 2.3	6.0 ± 0.6	20.5 ± 0.6	253.0 ± 9.3	11.0 ± 0.6	150.2 ± 15.4	2.6 ± 0.5

E_fl_: flexural modulus, σ_fl_: flexural strength, E_ten_: tensile modulus, σ_ten_: tensile strength, ε: elongation at break.

**Table 5:** The dependence of the residual linear particle size of CF on the content of the OPSU and PC.

Content	Arithmetic Average Fiber Length, µm	Weighted Average Fiber Length, µm
CF	220 ± 32	283 ± 26
PEI-CF	87 ± 11	102 ± 14
**OPSU**		
10%	118 ± 16	133 ± 11
15%	108 ± 14	126 ± 14
20%	125 ± 12	142 ± 17
**PC**		
10%	96 ± 10	113 ± 18
15%	106 ± 12	124 ± 15
20%	93 ± 11	108 ± 15

**Table 6:** Heat resistance of carbon-filled PEI with different content of OPSU and PC.

Content	T_2%_	T_5%_	T_10%_
PEI-CF	516	529	537
**OPSU**			
10%	500	528	539
15%	512	530	540
20%	510	529	539
**PC**			
10%	470	495	510
15%	474	508	518
20%	496	510	519

**Table 7:** Thermo-oxidative stability of carbon-filled PEI with different content of OPSU and PC.

Content	MFR at Various Exposure Times			
	4 min	5 min	30 min	60 min
PEI	13.2	12.7	12.6	11.3
PEI-CF	5.0	5.4	5.8	6.1
**OPSU**				
10%	6.7	7.0	6.4	6.4
15%	12.0	10.9	7.4	5.5
20%	15.6	14.4	8.7	5.8
**PC**				
10%	7.5	6.1	2.4	0.1
15%	10.3	10.0	3.7	1.2
20%	14.6	13.0	8.1	4.2

**Table 8:** Mechanical properties of the printed composites based on PEI/CF with OPSU and PC.

Properties	PEI/CF/OPSU	PEI/CF/PC
Flexural modulus, GPa	13.3 ± 1.2	8.4 ± 0.8
Flexural strength, MPa	227.6 ± 7.3	104.8 ± 6.4
Tensile modulus, GPa	5.4 ± 0.5	3.9 ± 0.2
Tensile strength, MPa	73.2 ± 13.2	73.0 ± 10.4
Elongation at break,%	2.2 ± 0.5	3.4 ± 1.6
